# High variability between regional histories of long-term atmospheric Pb pollution

**DOI:** 10.1038/s41598-020-77773-w

**Published:** 2020-12-01

**Authors:** Jack Longman, Vasile Ersek, Daniel Veres

**Affiliations:** 1 Marine Isotope Geochemistry, Institute for Chemistry and Biology of the Marine Environment (ICBM), University of Oldenburg, 26129 Oldenburg, Germany; 2 School of Geography and the Environment, University of Oxford, South Parks Road, Oxford, OX1 3QY UK; 3 Department of Geography and Environmental Sciences, Northumbria University, Newcastle-upon-Tyne, NE1 8ST UK; 4 Institute of Speleology, Romanian Academy, Clinicilor 5, 400006 Cluj-Napoca, Romania; 5 EDYTEM, Université Savoie Mont-Blanc, CNRS, Le Bourget du Lac, France

**Keywords:** Environmental chemistry, Environmental impact

## Abstract

The advent of metal processing was one of the key technological evolutions presaging the development of modern society. However, the interplay between metal use and the long-term changes it induced in the development and functioning of past societies remains unclear. We present a compilation of global records of anthropogenic atmospheric lead (Pb) spanning the last 4000 years, an effective indirect proxy for reliably assessing Pb emissions directly linked to human activities. Separating this global Pb pollution signal into regionally representative clusters allows identification of regional differences in pollution output that reflect technological innovations, market demands, or demise of various human cultures for last 4000 years. Our European reconstruction traces well periods of intensive metal production such as the Roman and Medieval periods, in contrast to clusters from the Americas, which show low levels of atmospheric Pb until the Industrial Revolution. Further investigation of the European synthesis results displays clear regional variation in the timing and extent of past development of polluting activities. This indicates the challenges of using individual reconstructions to infer regional or global development in Pb output and related pollution.

## Introduction

Since the dawn of early metal ages several millennia ago, humans have long altered the natural flux of heavy metals into the environment through activities such as mining, smelting and industrial processes which generate a variety of metals as pollution by-products^1^. Lead (Pb), a toxic, non-essential element, is a considerable metal pollutant in the environment, with anthropogenic Pb emissions comprising the majority of Pb flux to the atmosphere in the modern world^2^. Building on the pioneering work of Clair Patterson, who established the context and significance of historical pollution^3–5^, numerous studies have reconstructed past Pb pollution based on geochemical and isotopic analysis of paleoenvironmental records^6^. Due to its chemical immobility and limited biological uptake, Pb is effectively locked in place after deposition onto various sedimentary environments^7,8^. Geochemical and isotopic analyses may thus provide reliable reconstructions of changing levels and provenance shifts of atmospheric Pb through time. There are many examples of such work, utilizing peat bogs (e.g. Ref.^9^), lake sediments (e.g. Refs.^10–12^), ice cores (e.g. Refs.^13,14^) and other sedimentary archives (e.g. Ref.^15^). Such work has clearly proven the ability of this geochemical approach to reconstruct the history of atmospheric Pb deposition linked to changing pollution regimes. For example, many studies clearly identify down-core variations in Pb tracking known historical cultural and technological changes, with prominent increases in atmospheric Pb pollution associated with the advanced and metal-dependant Roman Empire observed across all European records^9,16^. Other studies provide strong constraints on the exact timing for the re-emergence of large-scale mining and smelting in the Middle Ages^17,18^. Alternatively, changing levels of Pb deposition may be indicative of shifts in smelting techniques employed, such as the shift towards amalgamation in South America from the 17th Century onward, resulting in lower Pb pollution release despite smelting of higher quantities of silver and gold ores^19,20^. More recently, variability in atmospheric Pb deposition may be linked to the burning of coal and other fossil fuels in the last two centuries^21^, and to the combustion of tetraethyl additives in gasoline^22^.

To date, however, apart from some regional syntheses^6,23,24^ no study has attempted to integrate the numerous globally distributed Pb records, which reconstruct historical metal use and changing industrial practices. As a result, each study is typically interpreted in isolation, or in combination with a small number of other studies. This is despite the reality of long-range transport of Pb^25–28^, meaning signals of major metallurgical activity should be present across a large number of sites (depending on atmospheric circulation patterns), in many instances providing a Pb pollution signal independent of local and regional variability in past human activities. A classic example is the identification of clear Roman and Medieval Pb pollution peaks in Scandinavian records^12,24^ or as far afield as Greenland ice^26^ and interpreted as long-range Pb input from continental and southern Europe. As such, to date it has been a challenge to assess whether variability in Pb pollution observed in a single record is regionally representative, or if that particular environmental archive records a purely local signal. Lead isotope provenance analysis may assist in solving such uncertainties, but Pb deposited within environmental records usually represents a cumulative signal from various emitting sources, often with similar isotopic ranges. The Pb isotopic signal must be considered an integrative proxy, allowing for example the separation of anthropogenic and geogenic Pb^17^. In Europe, the isotopic composition of various ores is well characterised, and from these data it is possible to constrain the signature of certain source areas^29^. However, geological studies indicate quite limited variability in Pb isotope signature of various ores within the major metallogenic provinces^30^, which to some extent impacts on reliably assessing provenance shifts in Pb pollution data.

Here we assemble a comprehensive long-term synthesis of atmospheric Pb pollution derived from available Pb concentration data in paleoenvironmental archives covering the past 4000 years (Table S1). Our work provides new insights into the past regional development of metal use and processing, and the environmental impact of such activities as reflected in long-term variability in atmospheric Pb fallout. Additionally, by grouping existing records into statistically well-assessed regional clusters, this approach provides us with a unique view on the development, extent, and level of metal use achieved by various societies through human history. Our approach can thus provide reliable comparison of environmental data in support of the often disparate and sometimes regionally not well-assessed archaeological information.

## Results and Discussion

### Pb pollution data as a proxy for the global history of mining, metallurgy and industry

**Figure 1:**
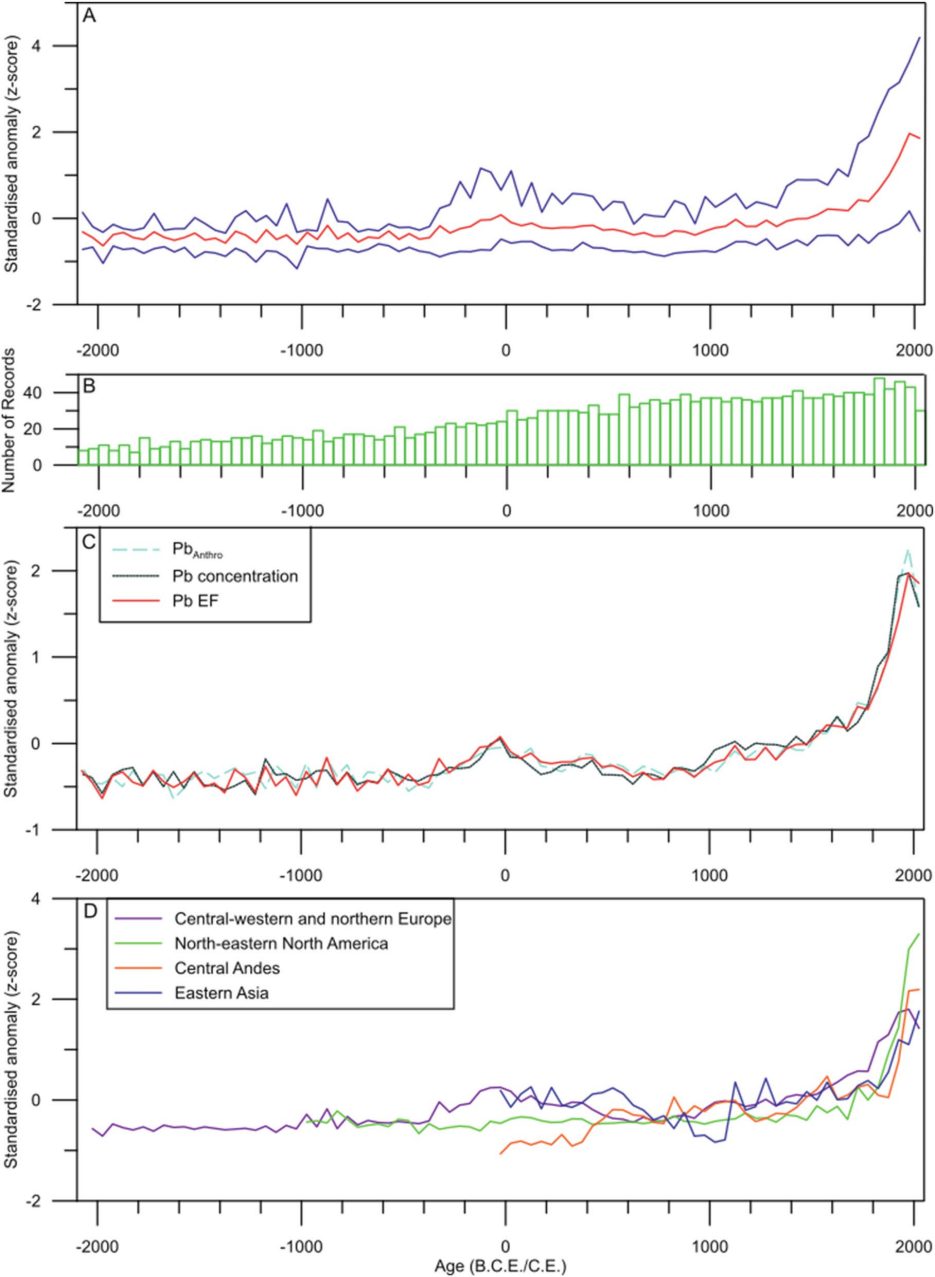
The global atmospheric Pb synthesis. (**A**) Displays the global synthesis of historical Pb pollution from 2000 BCE to present, as reconstructed from Pb enrichment factors (PbEF). The red line traces the mean value, while the two blue lines signify the 95% and 5% percentile value for the dataset. (**B**) Indicates the number of records used to for the synthesis in each bin. (**C**) Displays the mean values for syntheses developed from raw Pb concentration values for the period 2000 BCE to Present (dotted black line), Pb enrichment factors (PbEF, solid red line) and the anthropogenic fraction of Pb (Pb_Anthro_, dashed blue line); see text for details). (**D**) Displays the calculated continental syntheses, using PbEF values.

To investigate the global nature of the overall atmospheric Pb pollution synthesis (Fig. 1), we performed principal component analysis (PCA) on the binned z-score data from all 61 records analysed here. PCA results show the tight grouping of all but three of the analysed records, suggesting similar controls on variance for most data (Fig. S1). Two of these three studies are located in regions with no other data available (Serbia and north-western Canada; Refs.^8,11^), and likely represent regional signals not yet reconstructed elsewhere, but calling for a denser network of sites in order to better constrain the inferred regional variability in Pb pollution. The anomalous record from Bolivia^31^, shows extreme variability and so is most likely a record of a very local pollution signal. These three records have been removed from the global synthesis but data are used when discussing regional and local syntheses, where possible. This similarity in the grouping of the remaining studies is likely linked to concurrent global variability in atmospheric Pb levels, irrespective of the emitting sources. The scattering in grouping displays that each analyzed record also reflects at times the contribution of more local variability in Pb input. To better constrain this however, analysis would require a denser network of paleoenvironmental reconstructions of past Pb pollution, alongside deeper insights into regional to local archaeological data. Such an exercise displays the complexity of individual Pb records and demonstrates the challenge of reconstructing global trends from a series of records comprising local signal mixed in with a largely global one. As such, inferring global trends from individual records, unless they are located extremely distally from any local pollution (such as Greenland ice core records^32^), may lead to overinterpretation. However, by synthesizing across numerous records integrating across datasets comprising regionally representative pollution reconstructions, a ‘global’ Pb signal may be teased out (Fig. 1) which compares well with the low resolution Pb record from Greenland^32^, and with a more recent compilation of high-resolution Arctic records^33^. The non-global nature of this synthesis (zero, or a limited number of long-term records from key metallurgical locations such as Africa, the Middle East or South-Eastern Europe) suggests that integration of further records from such locations would be needed to better constrain this ‘global’ Pb pollution history.

**Figure 2:**
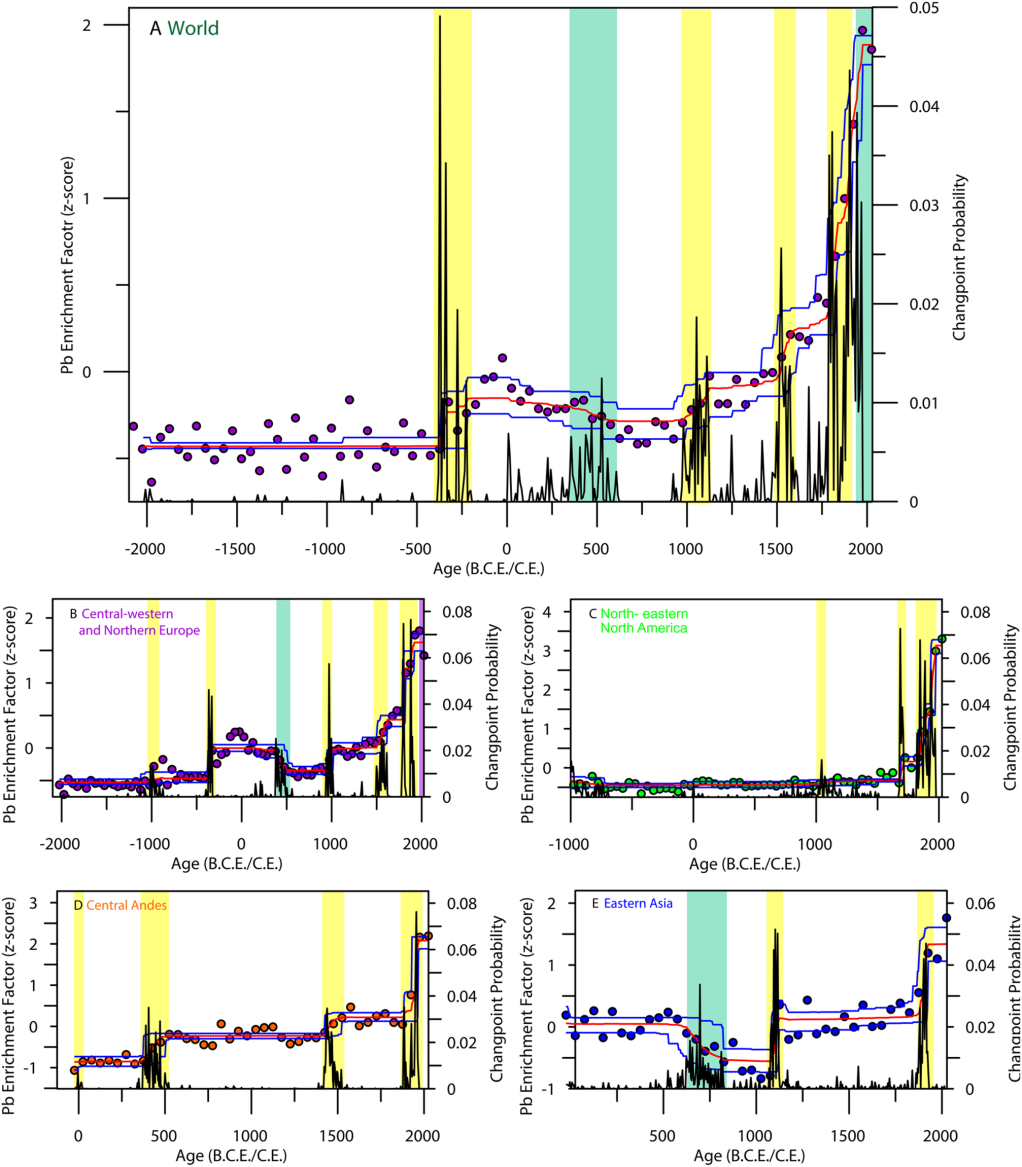
Changepoint modelling outputs for each synthesis. (**A**) Displays outputs from the full PbEF-derived synthesis. Coloured symbols signify mean values for each bin, with the model output is shown by a red line indicating the mean, and blue lines denoting 5% and 95% percentiles. Changepoints are indicated by the black line. Negative changepoints are highlighted by green rectangles and positive changepoints by yellow rectangles. (**B**–**E**) display the same as (**A**), but for the regional syntheses. Note changing age scales for each panel.

However, the variability seen in the synthesis of atmospheric Pb pollution follows well-understood past developments in mining and metal use as reconstructed from archaeological data (Figs. 1A, 2A). Between 2000 and 300 BCE, overall Pb levels are low in all records, which is to be expected as prior to the Eurasian Bronze Age, levels of anthropogenic metal pollution were low^34^ as metal processing focused mainly on smelting copper ores which may not lead to significant Pb pollution output^35^. Interestingly, there is no marked Pb enrichment throughout the Late Bronze Age and Early Iron Age (2000–1500 BCE) (Fig. 1A). This may reflect i., the small scale nature of worldwide smelting activities at this time when compared to later periods^34^, ii., a lack of Pb pollution data from regions known to have been at the forefront of metal processing at the time (e.g., South-Eastern Europe^17^, central Asia, India, northern Africa^36^); iii., the issue of widespread recycling of extant metal items at times rather than relying on primary extraction of ores as metal sources^37,38^, although it is possible these activities also produce Pb pollution in similar quantities to raw ore smelting^39^. Furthermore, the use of enrichment factors as a proxy for assessing levels of Pb pollution during the early metal ages may not be optimal, due to the rather small-scale nature of metal processing. Rather, shifts in isotopic composition may be a complementary approach, but it is beyond the scope of this study to consider both proxies, as most available Pb concentration records analysed here are not accompanied by Pb isotope data. We note, however, variability in our synthesis linked to the Late Bronze Age collapse, around 1200 BCE, that was a culturally disruptive period of disintegration and destruction of a number of major leading cultures throughout southern Eurasia^40^. This included the Mycenaeans, Kassites and Hittites, and severely affected trade routes in the wider Mediterranean region^40^. It is possible that the breakdown of trading routes caused a downfall in primary metal processing activity as established markets/cultures no longer received the necessary raw materials, or they lost the regional markets and incomes generated by metal production and trade^41^.

To infer when statistically significant shifts occur in the data, we model the likelihood of changepoints occurring (see “Methods”,^42^). Such an approach (Fig. 2) indicates that the first major increases in Pb pollution that had a global imprint occurred in line with the spread and strengthening of the Roman Republic and later Empire, and the contemporaneous peri-Mediterranean Classical Antiquity^43^. A sequence of changepoints denoting pollution increases (at c. 350, 275 and 225 BCE, Fig. 2A) well reflect Pb input from Iberian mining activity^16,32,44^, and the later intensification of Roman activity in locations such as Etruria and Sardinia^45^. High atmospheric Pb output throughout the peak of the Roman development is also well-recorded, before the Roman Empire’s eventual demise, documented in a series of changepoints indicating decreasing Pb pollution, starting from c.200 CE and with the largest at 425 CE (Fig. 2A). This tallies well with the chronology of Rome’s fall, with severe disruptions following the sacking of Rome in 410 CE, and the Imperial Crisis (235–284 CE)^46^.

Low atmospheric Pb levels between 600 and 1000 CE are related to the Dark Ages, a period of major technological regress across much of Europe. It was followed by the first in a series of changepoints relating to the steadily increasing Pb output denoting the onset of the technological innovation that characterized the last millennium in Europe (Fig. 2A). Four major changepoints, and numerous smaller ones, trace the increase in Pb pollution throughout Europe. The major changepoints at c. 1025 and 1510 CE are directly related to the re-discovery of mining and smelting techniques in central Europe during the Middle Ages as indicated by historical data^18^. These increases in Pb output can be linked to numerous innovations in the field of mining, from the introduction of water power to drain mines, new furnace technologies (including blast furnaces) and the development of cupellation^36^, all of which had a sizable impact on mining and metallurgical production, fostering the onset of later industrial scale mining activities. The series of changepoints from 1725 CE onwards are reflective of a number of worldwide advances in mining technology and industrial scale environmental pollution as the Industrial Revolution progressed^6^ and widespread coal burning began^21^. This is followed by the adoption of leaded gasoline, well-known to be the most considerable anthropogenic source of atmospheric Pb pollution in the 20th Century^47^. Noticeably, from about 1975 CE onwards, a clear negative changepoint is documented (Fig. 2A). This is representative of the impact of legislation banning leaded additives in gasoline, and the gradual decrease in atmospheric Pb levels as a result^47^.

### Assessing regional variability and representativeness of Pb reconstructions

As already demonstrated from PCA of the entire dataset (Fig. S1), individual Pb reconstructions are not necessarily fully representative of global signals, unless located in pristine environments far from proximal pollution sources. A more useful exercise therefore may be to group similarly located records, to infer regional changes in metal production, and for easier comparison to social complexity changes. The locations of the sites in this study lend themselves to attempting such grouping, with four regions well-represented: central-western and northern Europe, north-eastern North America, the central Andes, and eastern Asia (Fig. 1D). A small number of reconstructions fall outside of these regions (Table S1), and therefore are only considered in our reconstruction which includes all records.

For central-western and northern Europe our analysis relies on a regionally representative number of environmental records to accurately reconstruct trends in Pb pollution for the last 4000 years (Fig. 2), but fewer data are available for all other clusters, such that our reconstructions for the central Andes and eastern Asia only cover the last 2000 years, and north-eastern North America the last 3000 years (Fig. 2). Between 2000 and 1000 BCE atmospheric Pb levels in Europe were low (Fig. 2B), echoing the trend observed in the reconstruction from all records, of no significant Pb enrichment prior to Classical Antiquity (Fig. 2A). By 1000 BCE, atmospheric Pb in Europe shows steady increases. This is not seen in North America, reflecting the fact that the Iron Age and ensuing Classical Antiquity Pb pollution is a European/Near East feature, with the Roman Empire Pb pollution output being far more prominent than any other worldwide societies at this time (Fig. 2B)^6,10^. The lack of evidence for Pb pollution in North America until after 1000 CE reflects the lack of large-scale extractive metallurgy on the continent prior to this time. Considerable evidence of early extractive metallurgy on the continent stretching as far back as 6500 BCE is recorded as pollution in lake sediments^48^, but is primarily reflective of small-scale local smelting activities^10,49^.

There is no indication of an equivalent of Roman age Pb enrichment in South or North American paleopollution reconstructions. However, from roughly 500 CE onward, records from the central Andes trace similar (to the European synthesis), stepwise increases in Pb pollution (Fig. 2B–E). In the Andes, the earliest enrichment dates to roughly 400 CE, previously linked to rise of the pre-Incan Tiwanaku and Wari Empires^31^. The colonization of the Americas by Europeans from 1492 CE is well-reflected in increases in both North American and Andean Pb pollution at around this time (Fig. 2C,D). In North America, there is little evidence of pre-European use of base metals^50^, so low levels of anthropogenic Pb pollution prior to this point is to be expected. A small increase during the Mississippian period (1150–1450 CE) in the North American synthesis may be observed, reflective of limited pre-Colombian pollution on the continent^51,52^, represented by a low-probability changepoint from 1100 CE onwards (Fig. 2C). Positive changepoints in both datasets c.1460 CE however suggest that significant mining and metal smelting across the Americas pre-dates the arrival of Europeans^53^. The observed increase in anthropogenic Pb pollution in the central Andes is most likely to reflect the rise of the Inca, linked to a strong tradition of silver production^54^, as well as of other cultures throughout Mesoamerica.

All four regional clusters display a clear signal of Pb pollution related to the Industrial Revolution, and then that of the modern, highly polluting world (Fig. 2). The earliest changepoint is observed at c. 1625 CE in Europe, and may be related to new developments in mining and metallurgy during the seventeenth century, including those relating to the intensive iron smelting such as the cementation process (from 1619 CE onwards), the widespread appearance of bloomeries^55^, and the invention of the reverberatory furnace for (initially) lead smelting in the 1670s.

Changepoints at c. 1840 CE in North America and c. 1875 CE in South America are related to the adoption of polluting European technology in these locations^56^. The exact timing of these regional changepoints may be representative of the spread of industrial activity across the globe. Most recently, data from the 20th Century shows the ubiquitous nature of atmospheric Pb pollution (Fig. 2), as a result of the global use of leaded gasoline.

South-eastern Asia displays a clearly divergent record for the past 2000 years, with levels of Pb pollution comparable to the Roman Period in Europe (z-score ~ 0) for the period 0–500 CE (Fig. 2E), likely relating to the proficient mining culture of the Han Dynasty and other contemporaneous societies in the region^57^. The Pb pollution persisted until a sharp drop is observed after 500 CE followed by a series of changepoints from c. 650 CE (Fig. 2E). It is highly likely this drop, and the low levels of Pb pollution until after 1100 CE reflect economic disruptions related to military and political unrest in eastern Asia, including the An Lushan Rebellion against the ruling Tang dynasty between 755 and 763 CE, when up to 36 million people perished. The Chinese Tang dynasty was economically weakened by its reliance on borrowing^58^, and lost control of many provinces. By 907 CE the single ruling dynasty had devolved, leading to a period of prolonged political and societal division, with as many as 17 different state entities in what is now China^58^.

The return to greater atmospheric Pb enrichment in eastern Asia after 1150 CE traces well the economic development during the Yuan Dynasty that opened the first silver mines in Yunnan province around 1290 CE. Techniques such as cupellation were used to extract silver from local ores, alongside a considerable increase in iron production^59^. A preponderant Chinese source of Pb pollution during this period appears confirmed by the sudden decrease in pollution at roughly 1375 CE (Fig. 2E), corresponding to the demise of the Yuan Dynasty in 1368 CE, subsequent social instability, and the mining restrictions brought in by the proceeding Ming Dynasty^59^. Interestingly, there is no clear evidence of an industrial revolution in the corresponding Asian dataset until the second half of the twentieth century, perhaps a consequence of Imperial China’s and other regional powers failure to develop into industrialized economies at the time^60^.

### Intra-European variability

**Figure 3:**
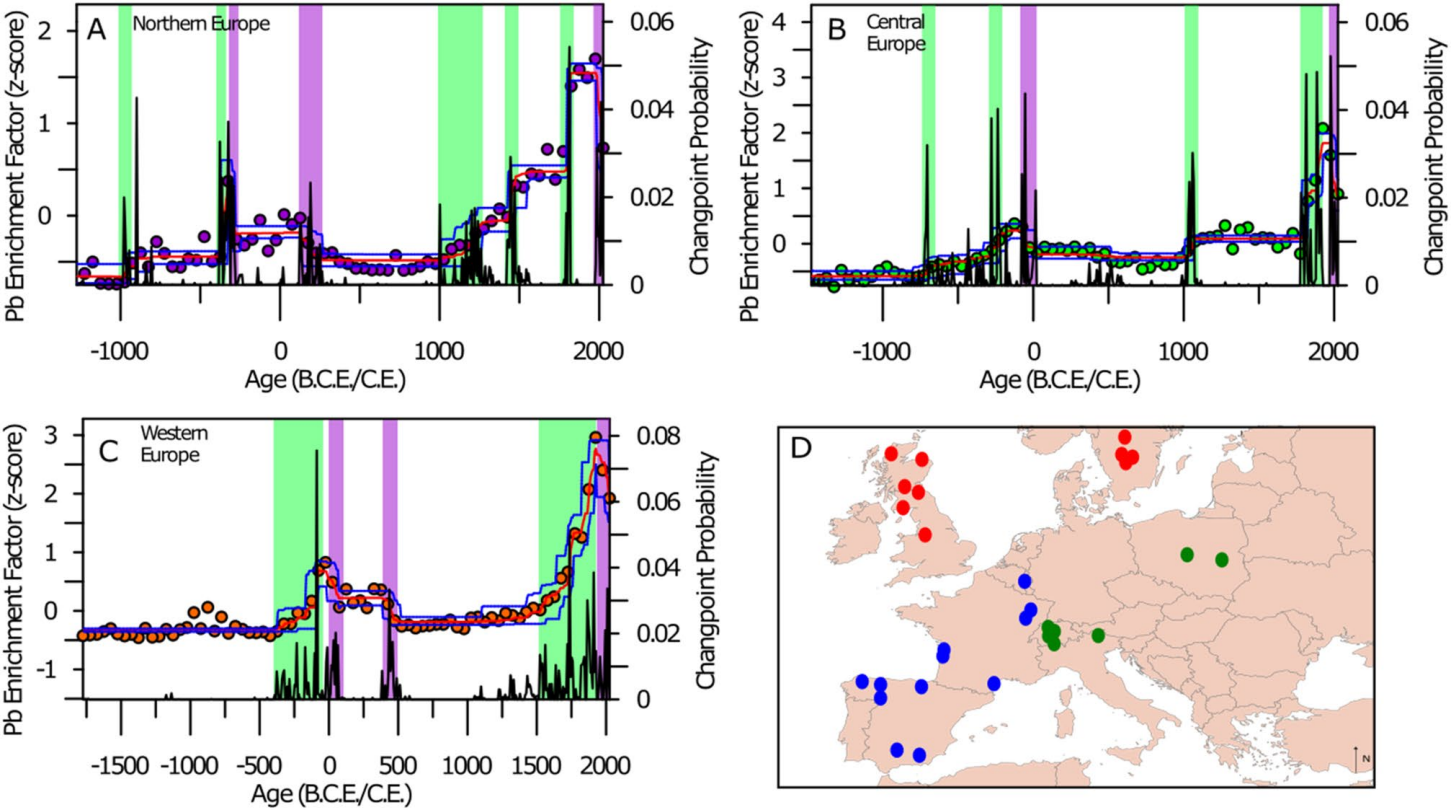
Changepoint modelling of European sub-regional syntheses. (**A**–**C**) Display changepoint modelling outputs from the PbEF-derived syntheses of considered European regions. Coloured points symbolise mean values for each bin, with the model output signified by a red line indicating the mean and blue lines denoting 5% and 95% percentiles. Changepoints are indicated by the black line. Negative changepoints are highlighted by purple rectangles and positive changepoints by green rectangles. (**B**–**E**) Display the same as (**A**), but for the regional syntheses. (**D**) is a map of Europe (created in ArcMap 10.3, Environmental Systems Resource Institute, ArcMap 10.3 ESRI, Redlands, California, http://desktop.arcgis.com/en/arcmap/), presenting the locations used to construct the syntheses, and the region they are included in, with red circles denoting northern sites, blue for western and green for central. Note changing age scales for each panel.

The availability of many European records allows for the reconstruction of Pb pollution history for much of the past 4000 years for western, central and northern Europe (Fig. 3), with the records contributing to each synthesis indicated in Fig. 3D. Our work in Europe is limited by the lack, apart from Ref.^17^, of long-term data for south-eastern Europe. This is despite the region comprising the rich Carpathian-Balkan mining fields, and likely crucial in terms of past metal processing in Europe, from the early metal ages onwards^61,62^.

From data in Fig. 3, the late Bronze Age and early Iron Age are the first positive changepoints seen in any records, but it appears Pb enrichment in western Europe may have begun prior to 4000 BCE, with z-score values in the west (~ − 0.31) greater than either central or northern Europe (both ~ − 0.65), suggesting Bronze Age metallurgy was more significant in western Europe than elsewhere (Fig. 3). Changepoints at c. 900 and 975 BCE in northern Europe indicate the onset of the Iron Age in this area, whilst in central Europe the first enrichment does not occur until c. 700 BCE (Fig. 3B).

This changepoint, and the subsequent gradual increase in Pb pollution in central Europe seems linked to the emergence of the Roman Empire, following the founding of Rome in 753 BCE, and the establishment of the Roman Republic in 509 BCE^63,64^. Changepoints denote the earliest imprint of Roman pollution in other regions, at c.325 BCE in northern (Fig. 3A), and c.375 BCE in western Europe (Fig. 3C). A second considerable increase in western Europe at c. 85 BCE may be the signature of Roman exploitation of Carthaginian mines following the Punic wars in the third and second centuries BCE. The high Pb pollution in all locations observed for the following two centuries is clearly linked to the apogee of the Roman Empire (Fig. 3). The earliest indication of the slow decline of the Roman Empire is seen in central European records, with two changepoints (at c. 50 and 25 CE) followed by a considerable drop in northern Europe at 125 CE and in western Europe at c.450 CE.

The Dark Ages are well-represented in records from all regions, with low levels of Pb pollution following the collapse of the Roman Empire persisting until c.1000 CE, when changepoints in both northern and central Europe, likely representative of the inception of Middle Age mining^33^, may be seen (Fig. 3A,B). The period 900–1200 CE was one of great population increases and technological advancement, especially with regards to mining. New ore discoveries, and the reopening of old mines, coupled to the application of Pb to the silver extraction method fuelled a boom, especially in central (Czech Republic and Germany) and northern (Great Britain) Europe^18,65^. No significant Pb increase is seen in records from western Europe until c. 1400 CE (Fig. 3C).

The imprint of the Industrial Revolution is also clear in all regions. Both northern and central Europe display clear changepoints at c. 1800 CE, which are related to the emergence of large-scale industrial pollution. There is a slight lag in the records from western Europe, with comparable enrichments not occurring until c. 1875 CE. In all reconstructions, the pattern of rising enrichment continues, with considerable leaded-gasoline related pollution throughout the 20th Century (Fig. 3). Final changepoints are observed in all records after 1975 CE, documenting major decreases in European Pb pollution. These are certainly related to the introduction of laws banning leaded gasoline and confirm the effectiveness of this policy in reducing atmospheric Pb pollution.

## Conclusions

We provide the first global synthesis of the levels of atmospheric lead (Pb) from 2000 BCE to present. This record confirms the coherence of palaeopollution records across Europe, North and South America, and therefore the long-range nature of anthropogenic Pb pollution. For the past 3000 years, it is possible to discern significant periods of Pb enrichment at times of enhanced known mining and metal processing activities (e.g. Roman Period, Medieval Period).

Significant inter-continental variability is also observed, again reflecting changing levels of development. For example, no equivalent of the European Roman Pb enrichment is observed in North America, and Asian records show a completely divergent trend to these in Europe. Finally, intra-continental variability is also observed, using Europe (as the best studied region) as an example. Here, smaller differences may be seen, for example the early onset of Roman pollution in central Europe, and the limited early Medieval pollution in western locations. This synthesis provides a valuable new look into the history of metal pollution on a global scale. It is, however, limited by the availability of data in some regions, with no records of sufficient quality from Africa or Australasia, and relatively few from outside Europe.

## Methods

We compiled 61 reconstructions of past atmospheric Pb pollution (Table S1, Fig. S2), with the selection criteria outlined in Supplementary Materials and Methods S1, resulting in a synthesis covering the past 4000 years. The compilation includes records from peat bogs, lake sediments, marshlands and ice cores (Table S1). Three geochemical parameters are presented here, raw Pb concentration (Pb_conc_), Pb enrichment factor (PbEF) and anthropogenic Pb fraction (Pb_Anthro_). The parameters PbEF and Pb_Anthro_ consider the eventual contribution of lithogenic (geogenic) Pb sources by normalising to the average amount of each normalizing conservative element (e.g. Sc, Zr, Ti) in the upper continental crust^66^. PbEF and Pb_Anthro_ thus provide a best estimate of the amount of anthropogenically sourced Pb^67^ identifiable within environmental records (See Supplementary Materials and Methods S1). To reduce the effect of inter-site variability and changing local geology, and to remove any bias toward sites located closer to pollution sources all data were converted to z-scores. These data were then binned at 50-year resolution (i.e. all data points within the ages 0–49 CE constitute bin ’25 CE’) to reduce biases toward reconstructions with greater temporal resolution, and averaged. For lower-resolution studies, no interpolation was used, and so the number of reconstructions represented in each bin decreases deeper in time, as data points are less numerous. Simply taking the 5^th^ and 95^th^ percentile of the raw z-score data is more representative of the variability in the datasets than using resampling methods. We tested this by comparing our reconstruction with one developed via Monte Carlo resampling, whereby 10,000 iterations of potential models from the raw mean and standard deviations were completed (Fig. S3). We believe such an approach acts to smooth the variability and leads to a simplified synthesis, and so we have not used it further in the overall analyses. Nevertheless, the Monte Carlo resampling results in very similar mean values (Fig. S3C).

To investigate the potential impact of record-related variability on the overall Pb synthesis, sensitivity tests were carried out. Binning at 100-year intervals reveals a very similar trend to 50 years at a lower resolution (Fig. S4), whilst binning at 25-year intervals leads to many bins containing too few data points. The type of archive used may also influence the reconstruction, but it is clear peat bogs and sediment records reconstruct the same trend as seen in the global Pb pollution synthesis, with ice core records diverging slightly, particularly in the first 500 years of the record from 2000 to 1500 BCE (Fig. S5). It is likely this is due to the resolution of ice core records, typically higher than 5 years/sample, which means that binning at 50-year resolution smooths out a large proportion of their data variability. This is evidenced by the similarity between a synthesis of all records with resolutions below 15 years/sample and the overall mean, whilst those with greater than 15 years/sample resolution showing slightly greater dissimilarity (Fig. S6).

Another crucial variable is the reliability of the age model and type of age-depth modelling used (e.g. Bayesian modelling, single spline, linear interpolation) in the original study. To investigate this, we have re-calculated age models using IntCal 13 radiocarbon calibration curve for Northern Hemisphere records^68^ and SHCal13 for Southern Hemisphere records^69^), alongside Bayesian age-depth modelling software (Bacon;^70^) for several records representing the variety of original age models and a variety of record types (Fig. S7). In all cases, the variation between original dating and our re-modelled ages is rather negligible, with 50-year bins smoothing out any discrepancies, resulting in very similar trends being represented (Fig. S7). In addition, several studies and particularly the ice cores rely on site-specific age models established using either annual layer counting and/or glaciological modelling^13,71^. As such, we use the original age-depth models as published, other than those which previously provided no age-depth models (e.g.^72^).

From the 61 reconstructions used, 35 are representative European palaeopollution, potentially skewing the global Pb pollution reconstruction. However, the reality of long-range Pb pollution has been repeatedly proven, with numerous examples, such as Roman Period Pb pollution having been clearly identified in Greenland ice^14,25^, inferences supported by Pb isotope provenance analysis (e.g. Ref.^26^). At 50-year resolution, utilizing normalized data, the overall synthesis should reconstruct global trends, with the representative Pb enrichment and/or Pb depletion periods discernible from background noise. The impact of local variability should also be considered, however, and therefore separate syntheses have been constructed using subsets of the overall database. Continental variability is investigated using records from four source areas: Central-western and Northern Europe, North-eastern North America, the Central Andes, and eastern Asia. These represent the regions of the globe with the most reconstructions available (Fig. S2). To investigate exactly when significant common and/or divergent changes are observed in the various records, we use changepoint modelling^42^, which statistically indicates when changes in the overall mean occur in the dataset.

PbEF data, a parameter which calculates the Pb enrichment above natural levels in any given record, has been used as the reference dataset for our reconstruction, since it encompasses anthropogenic Pb input, whilst allowing for the greatest number of reconstructions to be presented (Fig. 1A–D). This allows for the interpretation of variability linked solely to anthropogenic Pb rather than any detrital or erosional geogenic Pb, and circumvents issues relating to, for example, any bias from decomposition of organic matter within peat records. For calculating PbEF data, we normalize solely to the generally accepted values of targeted elements within the upper continental crust (UCC), as opposed to local rock composition to provide coherence across the dataset and allow for the greatest number of studies to be included. Furthermore, in a large number of studies, local rock data are unavailable. Where possible we employed the same normalising approach as the original publication.

## Supplementary information

Supplementary Information 1.

Supplementary Information 2.

## Data Availability

All data generated or analysed during this study are included in this published article (and its Supplementary Information files).
