# Supplementary material for: High variability between regional histories of long-term atmospheric Pb pollution
Source: Sci Rep. 2020 Dec 1;10:20890. doi: 10.1038/s41598-020-77773-w (PMC7708465; doi:10.1038/s41598-020-77773-w)
Supplement: Supplementary file 2 — Supplementary Information 2. [file 41598_2020_77773_MOESM2_ESM.pdf]

Supplementary Materials to "High variability between regional histories of long-term atmospheric Pb pollution"

Jack Longman,[a,b]* Vasile Ersek,[c] Daniel Veres,[d,e] *

[a] Marine Isotope Geochemistry, Institute for Chemistry and Biology of the Marine Environment (ICBM), University of Oldenburg, PO Box 2503, 26111 Oldenburg, Germany.

[b] School of Geography and the Environment, University of Oxford, South Parks Road, Oxford, OX1 3QY, UK

[c] Department of Geography and Environmental Sciences, Northumbria University, Newcastle-upon-Tyne, NE1 8ST, United Kingdom.

[d] Romanian Academy, Institute of Speleology, Clinicilor 5, 400006, Cluj-Napoca, Romania.

[e] EDYTEM, Université Savoie Mont-Blanc, CNRS, Le Bourget du Lac, France

*corresponding authors: jack.longman@uni-oldenburg.de; daniel.veres@ubbcluj.ro

Supplementary Methods and Materials

Selection Criteria for records

- Published prior to the end of 2018
- Must have a robust chronology, with at least five ^{14}C dates for the c. 4000-years of the reconstruction, and/or supplementary ^{210}Pb dating.
- Must be of sufficient length, stretching back to pre-anthropogenic conditions. Typically this is pre-Roman era, but in some locations (e.g. [1]), pre-anthropogenic conditions persisted much later. This allows for enrichment to be clearly observed, and for z-scores to consider what 'natural' conditions were.
- Data must be freely available in the publication, on a repository, or available from the authors.

We have limited the synthesis to only Pb, as other elements previously linked to anthropogenic pollution (e.g. Cu, Sn, Hg) either do not provide sufficient records to build reliable syntheses from, or are mobile in the environment, meaning reliable downcore chemical chronostratigraphies are difficult to attain [2,3]. Pb isotopes are not included in this work as their signatures vary from location to location, based upon shifting ore sources. As a result, there is no way of synthesising the variation across a number of different locations. At this stage of research we have also not included harbour-derived records despite their valuable contribution to our understanding of Pb paleopollution, as these are typically representative of purely local Pb pollution [4–6]. Further, harbour-derived archives are often an order of magnitude higher in concentration (>250ppm Pb) than other environmental records (e.g. ref.[6]), and so it is clear they are not recording atmospheric Pb pollution. As a result, such studies are hard to directly compare to those derived from environmental records where Pb pollution is typically derived solely from atmospheric deposition.

1.1 Extraction of anthropogenic signal

To ensure the reconstruction of the history of Pb pollution is made, and not simply a reconstruction of atmospheric Pb (natural and anthropogenic), two methods were used. The first is the calculation of enrichment factors (PbEFs). This approach normalises Pb pollution to the expected value of Pb and a conservative element (e.g. Ti, Sc, Zr) in the upper continental crust using the following equation:

$$EF = \frac{Pb_{sample}}{Zr_{sample}} / \frac{Pb_{UCC}}{Zr_{UCC}} \text{ (Eq. 1)}$$

The second approach is to calculate the anthropogenic fraction of Pb (Pb_{Anthro}).
Firstly, using the concentration of a conservative, lithogenic element (in this case Zirconium (Zr), and the expected composition of the upper continental crust (UCC)[7], the lithogenic fraction was calculated:

$$Pb_{Lithogenic} = Zr_{Sample} \times (Pb_{UCC} \times Zr_{UCC}) \text{ (Eq. 2)}$$

This value was subtracted from the overall Pb concentration of each sample to provide the likely anthropogenically derived Pb fraction:

$$Pb_{Anthropogenic} = Pb_{Sample} - Pb_{Lithogenic} \text{ (Eq. 3)}$$

Supplementary Figures

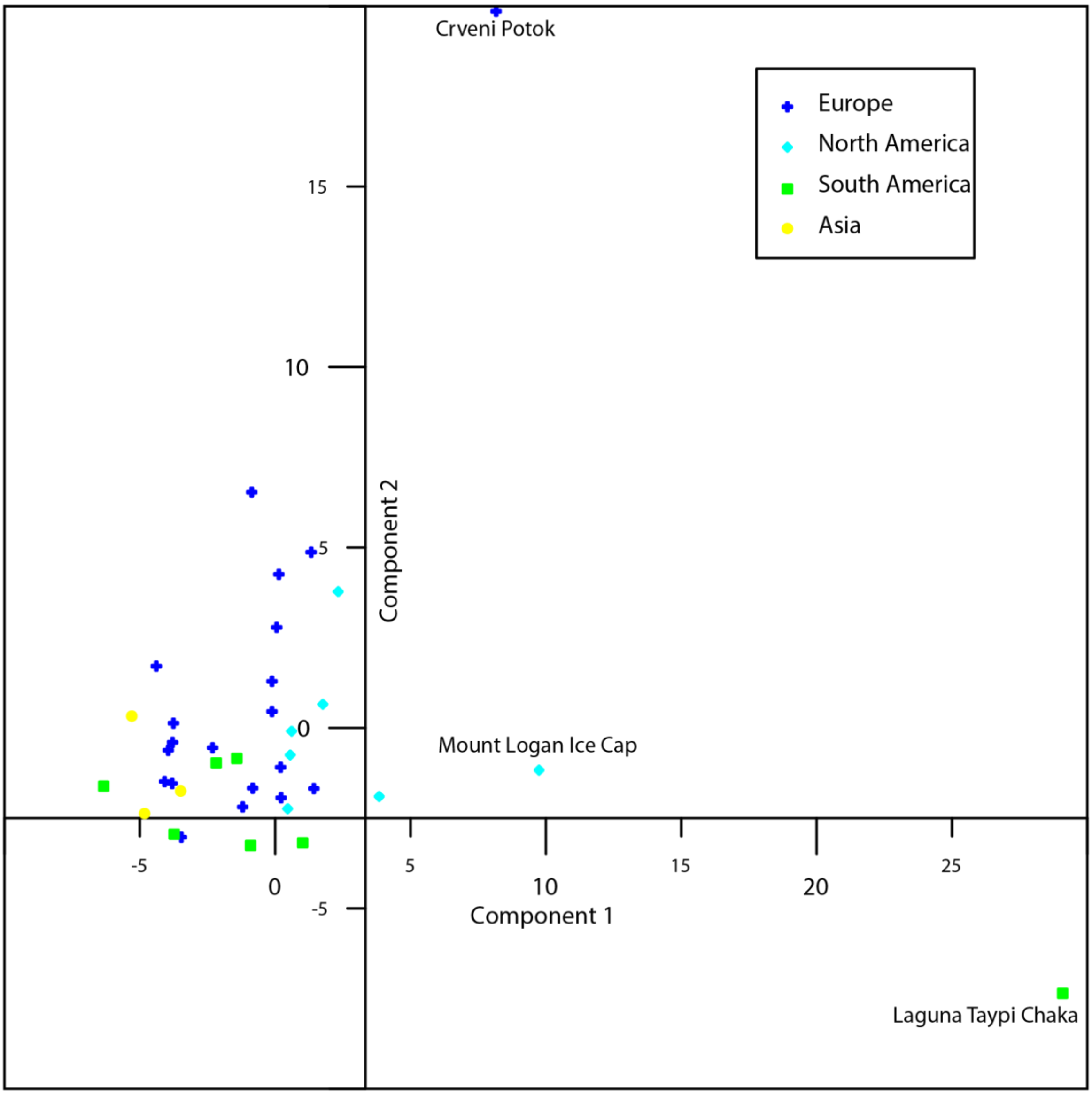

Fig. S1: Principal Component Analysis of all datasets used in this study. The vast majority of studies fall within a tight grouping, suggesting similar controls on their variance. Three studies plot outside of this grouping, suggesting different controls on their distribution and are highlighted; Crveni Potok [8]*, Mount Logan*[9] *and Laguna Taypi Chaka* [10].

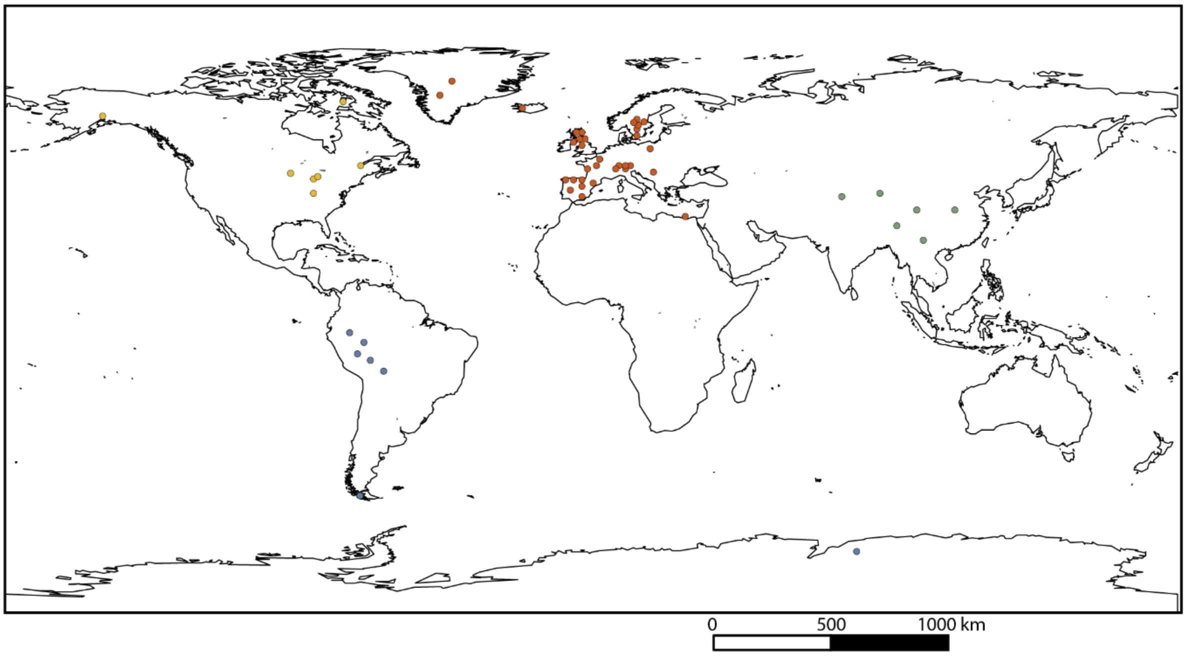

Fig. S2: Location of all sites mentioned in Supplementary Table 1. Each site is coloured according to its continental grouping; Central-western and Northern Europe (Orange), North-eastern North America (Yellow), Central Andes (Blue) and Eastern Asia (Green). Map created using World Vector Shorelines, GSGHH version 2.3.7, ref.[11].

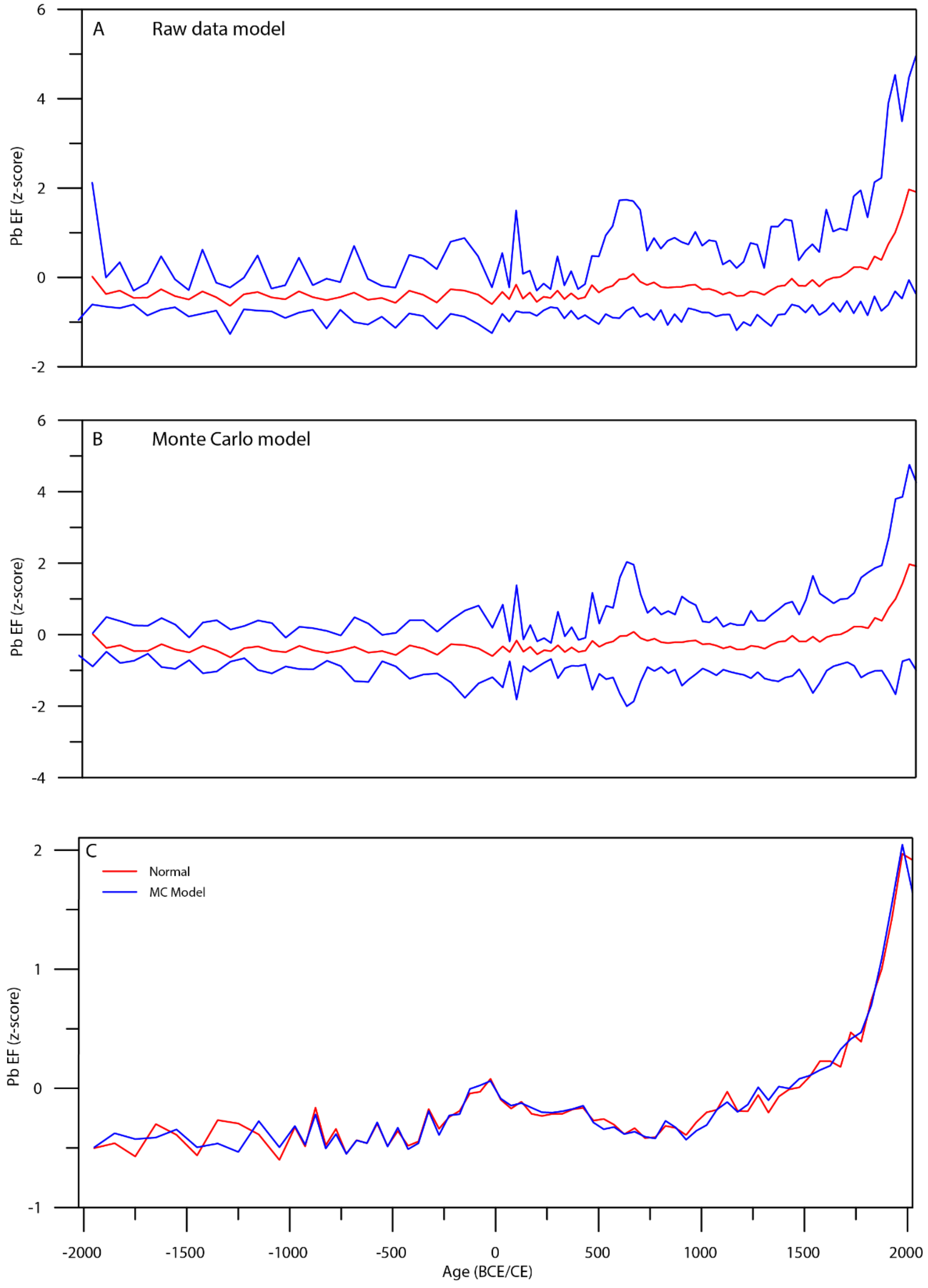

Fig. S3: Comparison of the Pb enrichment factor synthesis output using raw data (Panel A), and Monte Carlo-derived resampling data (Panel B). A comparison of the mean values from both approaches may be seen in panel C.

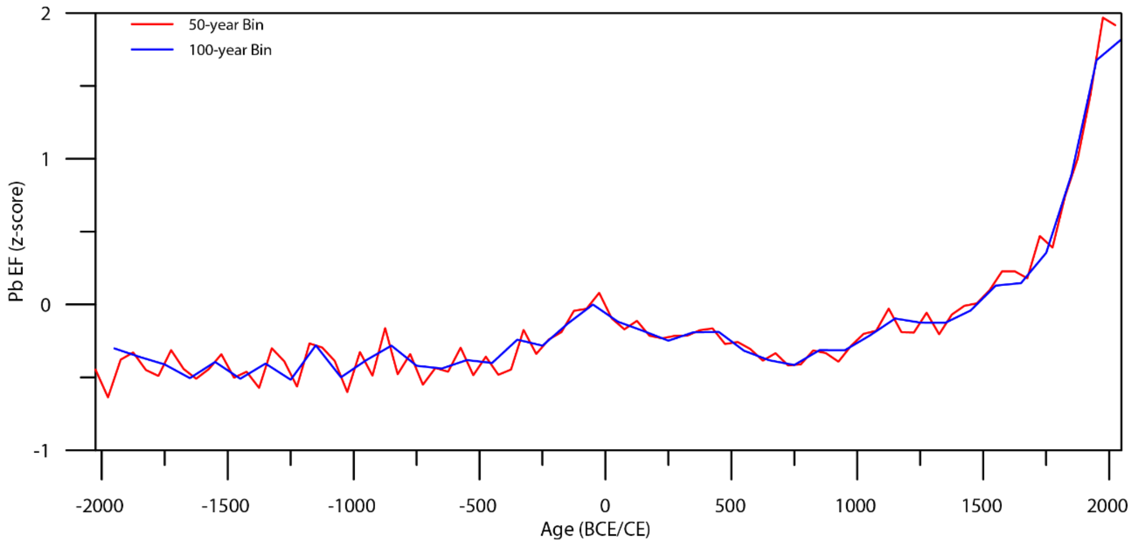

Fig. S4: Comparison of PbEF database when synthesis is carried out using 100-year (blue line) and 50-year (red line) binning.

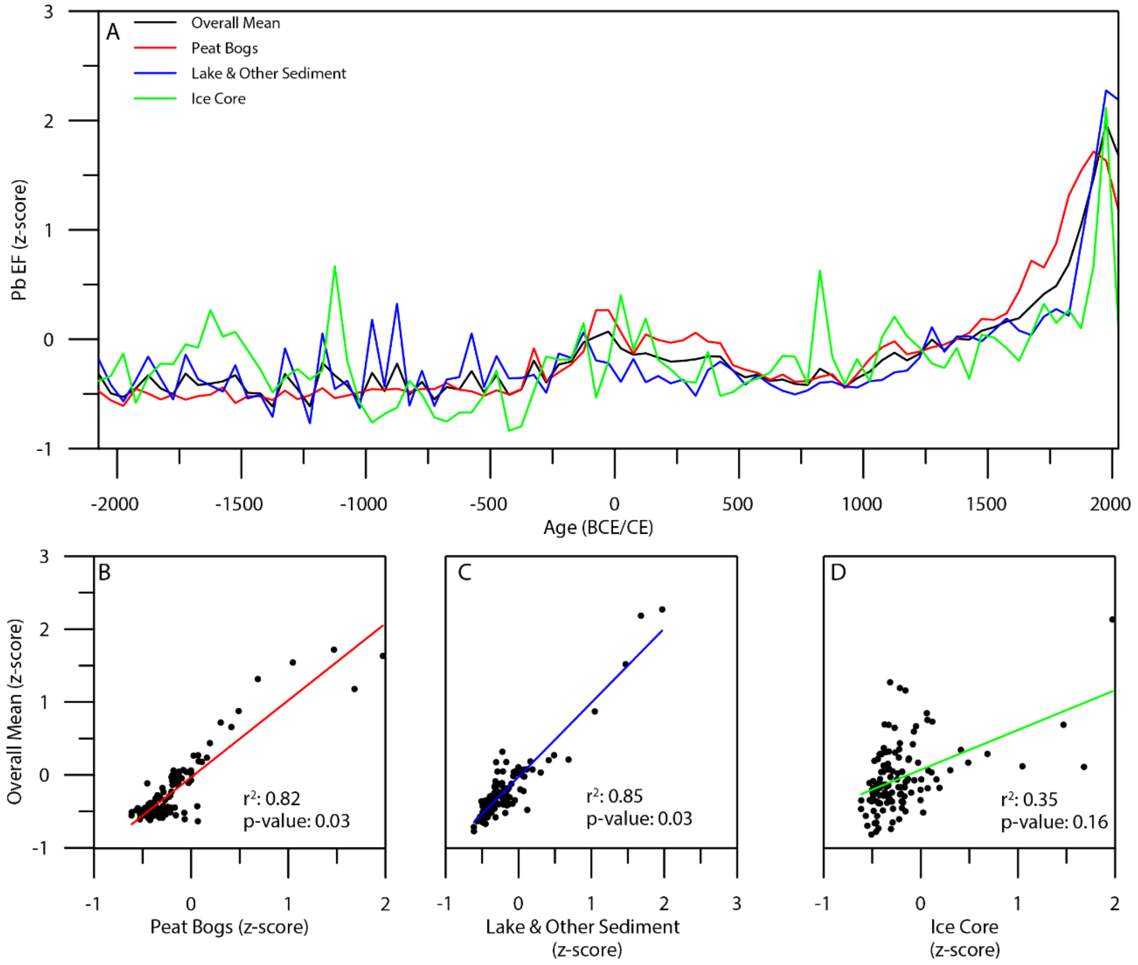

Fig. S5: (A) Comparison of mean values from syntheses carried out using records derived from just peat bogs (red line), lake/sediment cores (blue line) and ice cores (green line).

Panels C-D display scatter plots of each group versus the overall mean to determine similarity. Colours are as in panel A.

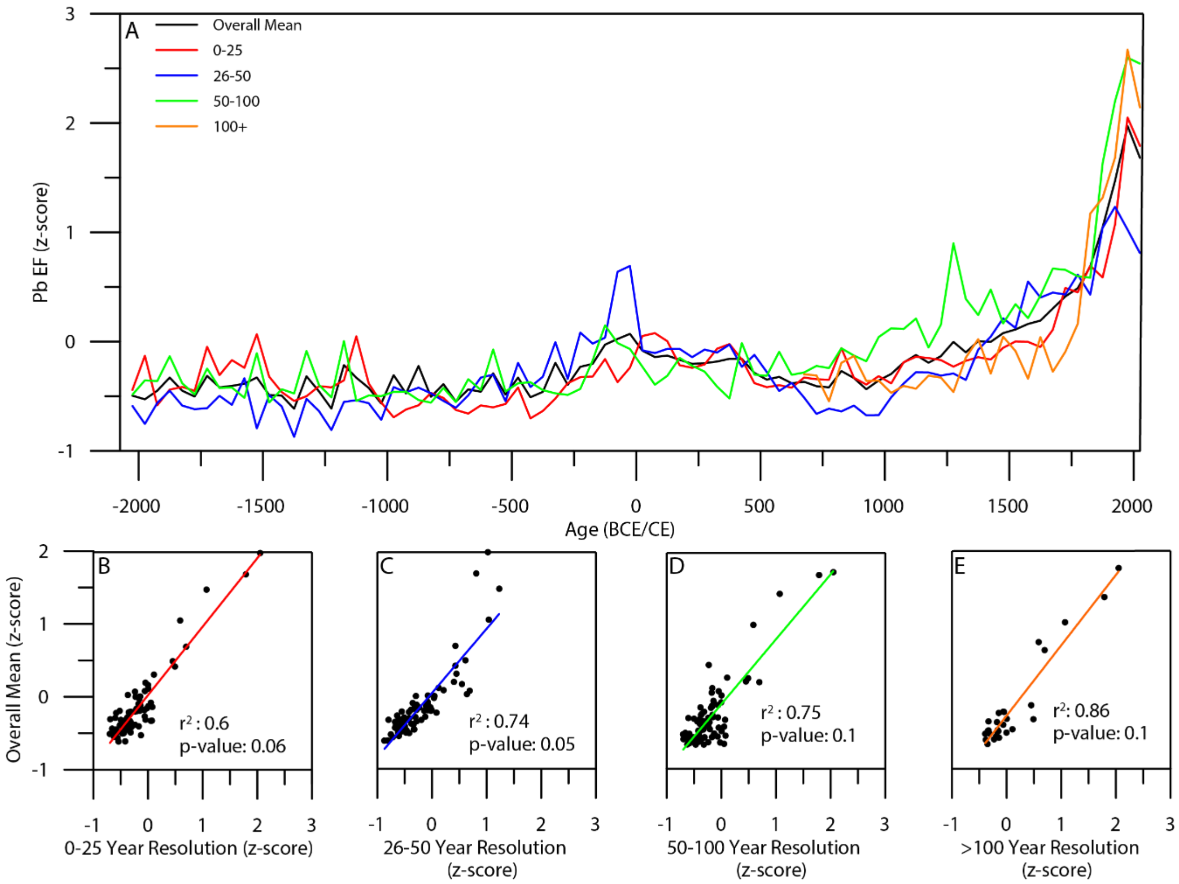

Fig. S6: (A) Comparison of mean values from syntheses carried out using records derived from records with resolutions of 0-25 yrs/sample (red line), 26-50 yrs/sample (blue line), 50-100 yrs/sample (green line) and >100 yrs/sample (orange line). Panels C-D display scatter plots of each group versus the overall mean to determine similarity. Colours are as in panel A.

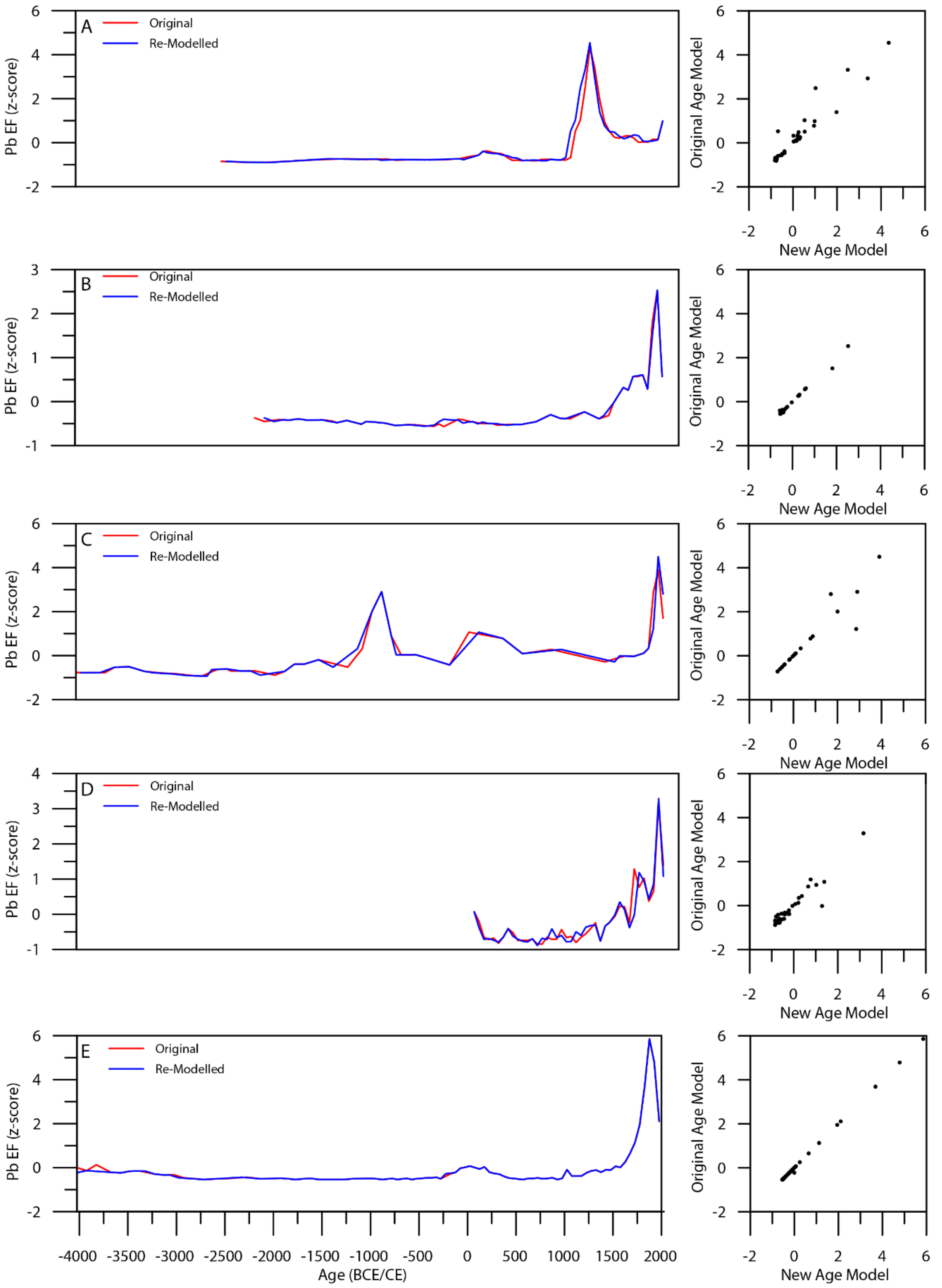

SI Figure 7: Comparison of PbEF records as displayed relative to the age model as originally published (red line), and to re-modelled ages (blue line). Scatter plots of the values

are displayed on the right of the figure. The record in Panel A is from [12]*, B from* [13]*, C from* [14]*, D from* [15] *and E from* [16]*.*

Supplementary Tables

Table S1: List of records used in this synthesis.

Record Number	Location Name	Latitude	Longitude	Country	Regional Cluster	European Cluster	Type of Record	Resolution (yrs/sample)	Reference
**1**	Colle Gnifetti	45.93	7.87	Italy	Central-western and northern Europe	Central	Ice	1.56	[17]
**2**	GRIP	72.57	-37.62	Greenland	Central-western and northern Europe	Northern	Ice	118.33	[18]
**3**	NGRIP 2	75.1	-42.32	Greenland	Central-western and northern Europe	Northern	Ice	0.99	[19]
**4**	Pierre Blanche Lagoon	43.5	3.86	France	Central-western and northern Europe	Western	Lagoon	30.42	[20]
**5**	Maryut Lagoon (Nile Delta)	31.15	29.9	Egypt	Not grouped	Not grouped	Lagoon	298.53	[21]
**6**	Laguna Roya	42.22	-6.77	Spain	Central-western and northern Europe	Western	Lake	43.39	[12]
**7**	Lake Lucerne	47.05	8.58	Switzerland	Central-western and northern Europe	Central	Lake	13.52	[22]
**8**	Loch Laxford	58.37	-5	United Kingdom	Central-western and northern Europe	Northern	Lake	7.22	[1]
**9**	Longemer Lake	48.07	6.95	France	Central-western and northern Europe	Western	Lake	55.64	[23]
**10**	Meidsee	46.22	7.67	Switzerland	Central-western and	Central	Lake	59.92	[22]

					northern Europe				
**11**	La Perge marsh	45.3	-1	France	Central-western and northern Europe	Western	Marsh	136.84	[24]
**12**	St. Ciers-sur-Gironde marsh	45.4	-1	France	Central-western and northern Europe	Western	Marsh	246.39	(20)
**13**	Viðarhólmi salt marsh	64.8	-22.4	Iceland	Central-western and northern Europe	Northern	Marsh	14.14	[25]
**14**	Carsegowan Moss	55.2	-2.72	United Kingdom	Central-western and northern Europe	Northern	Peat	30.62	[26]
**15**	Coltrondo	46.66	12.45	Italy	Central-western and northern Europe	Central	Peat	53.33	[27]
**16**	Crveni Potok	43.91	19.42	Serbia	Central-western and northern Europe	Not Grouped	Peat	51.88	[8]
**17**	Dumme Moss	57.5	14.03	Sweden	Central-western and northern Europe	Northern	Peat	143.62	[28]
**18**	Etang de la Gruyere	47.24	7.05	Switzerland	Central-western and northern Europe	Central	Peat	121.15	[29]
**19**	Flanders Moss	56.15	-4.2	United Kingdom	Central-western and northern Europe	Northern	Peat	22.42	[30]
**20**	Kohlhutte Moor	48.06	8.18	Germany	Central-western and northern Europe	Central	Peat	39.52	[31]
**21**	La Molina mire	43.38	-6.33	Spain	Central-western and northern Europe	Western	Peat	18.51	[32]
**22**	La Molina mire	43.38	-6.33	Spain	Central-western and	Western	Peat	37.98	[33]

					northern Europe				
**23**	Laguna de Río Seco	37.05	-3.35	Spain	Central-western and northern Europe	Western	Peat	140.32	34
**24**	Lindow bog	53.32	-2.27	United Kingdom	Central-western and northern Europe	Northern	Peat	37.52	35
**25**	Misten bog	50.56	6.16	Belgium	Central-western and northern Europe	Western	Peat	30.88	36
**26**	Onneby	60.12	13.03	Sweden	Central-western and northern Europe	Northern	Peat	186.97	37
**27**	Pena da Cadela	43.57	-7.57	Spain	Central-western and northern Europe	Western	Peat	61.05	38
**28**	Penido Vello	43.37	-7.43	Spain	Central-western and northern Europe	Western	Peat	68.01	16
**29**	Radzyn	53.38	18.93	Poland	Central-western and northern Central-western and northern Europe	Central	Peat	19.95	39
**30**	Red Moss of Balerno	55.85	-3.33	United Kingdom	Central-western and northern Europe	Northern	Peat	33.91	26
**31**	Rywałd	53.37	19.05	Poland	Central-western and northern Europe	Central	Peat	24.2	39
**32**	Store Moss	57.25	13.92	Sweden	Central-western and northern Europe	Northern	Peat	130.17	37
**33**	Tranerödsmosse	56.05	13.18	Sweden	Central-western and northern Europe	Northern	Peat	139.8	28

**34**	Trolls	15.67	14.5	Sweden	Central-western and northern Europe	Northern	Peat	203.12	37
**35**	Turclossie Moss	57.62	-2.18	United Kingdom	Central-western and northern Europe	Northern	Peat	28.59	26
**36**	Devon Island	75	-82	Canada	North-eastern North America		Ice	126.61	40
**37**	Mount Logan	60.57	-140.2	Canada	Not grouped		Ice	24.89	9
**38**	Copper Falls Lake	47.42	-88.19	United States	North-eastern North America		Lake	69.33	41
**39**	Horseshoe	38.7	-90.1	United States	North-eastern North America		Lake	103.98	42
**40**	Lake Manganese	47.5	-87.9	United States	North-eastern North America		Lake	64.68	41
**41**	Lake Medora	47.4	-88	United States	North-eastern North America		Lake	66.04	41
**42**	Lake Pepin 97	44.49	92.3	United States	North-eastern North America		Lake	137.24	43
**43**	Lake Pepin D2	44.49	92.3	United States	North-eastern North America		Lake	29.56	43
**44**	McCargoe Cove	48.1	-88.7	United States	North-eastern North America		Lake	16.71	44
**45**	Point d'Escuminac	46.92	-65.5	Canada	North-eastern North America		Peat	16.15	1
**46**	Law Dome	-66.73	112.83	Antarctica	Not grouped		Ice	196.64	45
**47**	Quelccaya	-13.9	-70.9	Peru	Central Andes		Ice	4.98	46
**48**	Illimani	-16	-67	Bolivia	Central Andes		Ice	9.95	47
**49**	Laguna Lobato	-19	-65.68	Bolivia	Central Andes		Lake	32.44	10

		.63						
**50**	Laguna Pirhuacocoha	-11.52	-76.07	Peru	Central Andes	Lake	11.03	[10]
**51**	Laguna Taypi Chaka	-16.22	-68.35	Bolivia	Central Andes	Lake	82.19	[10]
**52**	Sajama	-18.1	-68.88	Bolivia	Central Andes	Lake	1154.2	[48]
**53**	Karukinka	-53.87	-69.58	Chile	Central Andes	Peat	64.03	[13]
**54**	Puruogangri ice cap	33.92	89.08	China	Eastern Asia	Ice	0.99	[49]
**55**	Hongyuan	32.76	102.52	China	Eastern Asia	Peat	102.91	[50]
**56**	Jinchuan	42.36667	126.43	China	Eastern Asia	Peat	12.274	[51]
**57**	Dian A-12	24.7	104.68	China	Eastern Asia	Lake	74.54	[52]
**58**	Dian F-14	24.7	104.68	China	Eastern Asia	Lake	68.99	[52]
**59**	Lake Erhai	28.78	100.18	China	Eastern Asia	Lake	53.41	[53]
**60**	Lake Lianghzhi	30.25	114.47	China	Eastern Asia	Lake	249.83	[54]
**61**	Xing Yun	24.17	102.77	China	Eastern Asia	Lake	47.91	[55]

1. Kylander, M. E., Weiss, D. J. & Kober, B. Two high resolution terrestrial records of atmospheric Pb deposition from New Brunswick, Canada, and Loch Laxford, Scotland. *Sci. Total Environ.* **407**, 1644–1657 (2009).
2. Novak, M. *et al.* Experimental Evidence for Mobility/Immobility of Metals in Peat. *Environ. Sci. Technol.* **45**, 7180–7187 (2011).
3. Rausch, N. *et al.* Comparison of atmospheric deposition of copper, nickel, cobalt, zinc, and cadmium recorded by Finnish peat cores with monitoring data and emission records. *Environ. Sci. Technol.* **39**, 5989–5998 (2005).

4. Delile, H., Blichert-Toft, J., Goiran, J.-P., Keay, S. & Albarede, F. Lead in ancient Rome's city waters. *Proc. Natl. Acad. Sci.* **111**, 6594–6599 (2014).
5. Delile, H. *et al.* A lead isotope perspective on urban development in ancient Naples. *Proc. Natl. Acad. Sci. U. S. A.* **113**, 6148–6153 (2016).
6. Delile, H. *et al.* Economic resilience of Carthage during the Punic Wars: Insights from sediments of the Medjerda delta around Utica (Tunisia). *Proc. Natl. Acad. Sci. U. S. A.* **116**, 9764–9769 (2019).
7. Wedepohl, K. H. The composition of the continental crust. *Geochim. Cosmochim. Acta* **59**, 1217–1232 (1995).
8. Longman, J., Veres, D., Finsinger, W. & Ersek, V. Exceptionally high levels of lead pollution in the Balkans from the Early Bronze Age to the Industrial Revolution. *Proc. Natl. Acad. Sci. U. S. A.* **115**, E5661–E5668 (2018).
9. Osterberg, E. *et al.* Ice core record of rising lead pollution in the North Pacific atmosphere. *Geophys. Res. Lett.* **35**, L05810 (2008).
10. Cooke, C. A., Abbott, M. B. & Wolfe, A. P. Late-Holocene atmospheric lead deposition in the Peruvian and Bolivian Andes. *The Holocene* **18**, 353–359 (2008).
11. Wessel, P. & Smith, W. H. F. A global, self-consistent, hierarchical, high-resolution shoreline database. *J. Geophys. Res. B Solid Earth* **101**, 8741–8743 (1996).
12. Hillman, A. L. *et al.* Lead pollution resulting from Roman gold extraction in northwestern Spain. *The Holocene* 095968361769390 (2017) doi:10.1177/0959683617693903.
13. De Vleeschouwer, F. *et al.* Emissions from Pre-Hispanic Metallurgy in the South American Atmosphere. *PLoS One* **9**, e111315 (2014).
14. García-Alix, A. *et al.* Anthropogenic impact and lead pollution throughout the Holocene in Southern Iberia. *Sci. Total Environ.* **449**, (2013).
15. Marshall, W. A., Clough, R. & Gehrels, W. R. The isotopic record of atmospheric lead fall-out on an Icelandic salt marsh since AD 50. *Sci. Total Environ.* **407**, 2734–2748 (2009).
16. Kylander, M. E. *et al.* Refining the pre-industrial atmospheric Pb isotope evolution curve in Europe using an 8000 year old peat core from NW Spain. *Earth Planet. Sci. Lett.* **240**, 467–485 (2005).
17. More, A. F. *et al.* Next-generation ice core technology reveals true minimum natural levels of lead (Pb) in the atmosphere: Insights from the Black Death. *GeoHealth* (2017) doi:10.1002/2017GH000064.
18. Rosman, K. J. R., Chisholm, W., Hong, S., Candelone, J. P. & Boutron, C. F. Lead from Carthaginian and Roman Spanish mines isotopically identified in Greenland ice dated from 600 B.C. to 300 A.D. *Environ. Sci. Technol.* **31**, 3413–3416 (1997).
19. McConnell, J. R. *et al.* Lead pollution recorded in Greenland ice indicates European emissions tracked plagues, wars, and imperial expansion during antiquity. *Proc. Natl. Acad. Sci. U. S. A.* **115**, 5726–5731 (2018).
20. Elbaz-Poulichet, F., Dezileau, L., Freydier, R., Cossa, D. & Sabatier, P. A 3500-Year Record of Hg and Pb Contamination in a Mediterranean Sedimentary Archive (The Pierre Blanche Lagoon, France). *Environ. Sci. Technol.* **45**, 8642–8647 (2011).
21. Véron, A. J. *et al.* A 6000-year geochemical record of human activities from Alexandria (Egypt). *Quat. Sci. Rev.* **81**, 138–147 (2013).
22. Thevenon, F., Guédron, S., Chiaradia, M., Loizeau, J. L. & Poté, J. (Pre-) historic changes in natural and anthropogenic heavy metals deposition inferred from two contrasting Swiss Alpine lakes. *Quat. Sci. Rev.* **30**, 224–233 (2011).
23. Mariet, A.-L. *et al.* Tracking past mining activity using trace metals, lead isotopes and compositional data analysis of a sediment core from Longemer Lake, Vosges

Mountains, France. *J. Paleolimnol.* **60**, 399–412 (2018).
24. Alfonso, S., Grousset, F., Massé, L. & Tastet, J.-P. A European lead isotope signal recorded from 6000 to 300 years BP in coastal marshes (SW France). *Atmos. Environ.* **35**, 3595–3605 (2001).
25. Marshall, W. a, Clough, R. & Gehrels, W. R. The isotopic record of atmospheric lead fall-out on an Icelandic salt marsh since AD 50. *Sci. Total Environ.* **407**, 2734–48 (2009).
26. Cloy, J. M., Farmer, J. G., Graham, M. C., Mackenzie, A. B. & Cook, G. T. Historical records of atmospheric Pb deposition in four Scottish ombrotrophic peat bogs: An isotopic comparison with other records from western Europe and Greenland. (2008) doi:10.1029/2007GB003059.
27. Segnana, M. *et al.* Holocene vegetation history and human impact in the eastern Italian Alps: a multi-proxy study on the Coltrondo peat bog, Comelico Superiore, Italy. *Veg. Hist. Archaeobot.* (2019) doi:10.1007/s00334-019-00749-y.
28. Klaminder, J., Renberg, I., Bindler, R. & Emteryd, O. Isotopic trends and background fluxes of atmospheric lead in northern Europe: Analyses of three ombrotrophic bogs from south Sweden. *Global Biogeochem. Cycles* **17**, 19–1 (2003).
29. Shotyk, W. *et al.* History of Atmospheric Lead Deposition Since 12,370 14C yr BP from a Peat Bog, Jura Mountains, Switzerland. *Science (80-.).* **281**, 1635–1640 (1998).
30. Cloy, J. M., Farmer, J. G., Graham, M. C., MacKenzie, A. B. & Cook, G. T. A comparison of antimony and lead profiles over the past 2500 years in Flanders Moss ombrotrophic peat bog, Scotland. in *Journal of Environmental Monitoring* vol. 7 1137–1147 (The Royal Society of Chemistry, 2005).
31. Le Roux, G. *et al.* Recent atmospheric Pb deposition at a rural site in southern Germany assessed using a peat core and snowpack, and comparison with other archives. *Atmos. Environ.* **39**, 6790–6801 (2005).
32. Martínez Cortizas, A., López-Merino, L., Bindler, R., Mighall, T. & Kylander, M. Atmospheric Pb pollution in N Iberia during the late Iron Age/Roman times reconstructed using the high-resolution record of La Molina mire (Asturias, Spain). *J. Paleolimnol.* **50**, 71–86 (2013).
33. Martínez-Cortizas, A., López-Merino, L., Bindler, R., Mighall, T. & Kylander, M. E. Early atmospheric metal pollution provides evidence for Chalcolithic/Bronze Age mining and metallurgy in Southwestern Europe. *Sci. Total Environ.* **545–546**, 398–406 (2016).
34. García-Alix, a. *et al.* Anthropogenic impact and lead pollution throughout the Holocene in Southern Iberia. *Sci. Total Environ.* **449**, 451–460 (2013).
35. Le Roux, G. *et al.* Identifying the sources and timing of ancient and medieval atmospheric lead pollution in England using a peat profile from Lindow bog, Manchester. *J. Environ. Monit.* **6**, 502–510 (2004).
36. De Vleeschouwer, F. *et al.* Anthropogenic impacts in North Poland over the last 1300years — A record of Pb, Zn, Cu, Ni and S in an ombrotrophic peat bog. *Sci. Total Environ.* **407**, 5674–5684 (2009).
37. Brännvall, M. L. *et al.* The medieval metal industry was the cradle of modern large-scale atmospheric lead pollution in northern Europe. *Environ. Sci. Technol.* **33**, 4391–4395 (1999).
38. Martínez Cortizas, A. *et al.* Atmospheric Pb deposition in Spain during the last 4600 years recorded by two ombrotrophic peat bogs and implications for the use of peat as archive. *Sci. Total Environ.* **292**, 33–44 (2002).
39. Babos, H. B. *et al.* Evidence for the onset of mining activities during the 13th century

in Poland using lead isotopes from lake sediment cores. *Sci. Total Environ.* **683**, 589–599 (2019).
40. Zheng, J., Shotyk, W., Krachler, M. & Fisher, D. A. A 15,800-year record of atmospheric lead deposition on the Devon Island Ice Cap, Nunavut, Canada: Natural and anthropogenic enrichments, isotopic composition, and predominant sources. *Global Biogeochem. Cycles* **21**, n/a-n/a (2007).
41. Pompeani, D. P., Abbott, M. B., Steinman, B. A. & Bain, D. J. Lake Sediments Record Prehistoric Lead Pollution Related to Early Copper Production in North America. *Environ. Sci. Technol.* **47**, 5545–5552 (2013).
42. Pompeani, D. P. *et al.* The environmental impact of a pre-Columbian city based on geochemical insights from lake sediment cores recovered near Cahokia. *Quat. Res.* (2019) doi:10.1017/qua.2018.141.
43. Dean, W. Holocene Record of Major and Trace Components in the Sediments of an Urban Impoundment on the Mississippi River: Lake Pepin, Minnesota and Wisconsin. *USGS Open File Rep.* (2009).
44. Pompeani, D. P., Abbott, M. B., Bain, D. J., DePasqual, S. & Finkenbinder, M. S. Copper mining on Isle Royale 6500–5400 years ago identified using sediment geochemistry from McCargoe Cove, Lake Superior. *The Holocene* **25**, 253–262 (2015).
45. Vallelonga, P. *et al.* Lead, Ba and Bi in Antarctic Law Dome ice corresponding to the 1815 AD Tambora eruption: An assessment of emission sources using Pb isotopes. *Earth Planet. Sci. Lett.* **211**, 329–341 (2003).
46. Uglietti, C., Gabrielli, P., Cooke, C. A., Vallelonga, P. & Thompson, L. G. Widespread pollution of the South American atmosphere predates the industrial revolution by 240 y. *Proc. Natl. Acad. Sci.* **112**, 2349–2354 (2015).
47. Eichler, A., Gramlich, G., Kellerhals, T., Tobler, L. & Schwikowski, M. Pb pollution from leaded gasoline in South America in the context of a 2000-year metallurgical history. *Sci. Adv.* **1**, 8 (2015).
48. Hong, S. *et al.* Atmospheric heavy metals in tropical South America during the past 22 000 years recorded in a high altitude ice core from Sajama, Bolivia. *J. Environ. Monit.* **6**, 322–326 (2004).
49. Beaudon, E., Gabrielli, P., Sierra-Hernández, M. R., Wegner, A. & Thompson, L. G. Central Tibetan Plateau atmospheric trace metals contamination: A 500-year record from the Puruogangri ice core. *Sci. Total Environ.* **601**–**602**, 1349–1363 (2017).
50. Ferrat, M. *et al.* Lead atmospheric deposition rates and isotopic trends in Asian dust during the last 9.5kyr recorded in an ombrotrophic peat bog on the eastern Qinghai-Tibetan Plateau. *Geochim. Cosmochim. Acta* **82**, 4–22 (2012).
51. Guo, B., Wang, J., Lin, C., He, M. & Ouyang, W. Anthropogenic and lithogenic fluxes of atmospheric lead deposition over the past 3600 years from a peat bog, Changbai Mountains, China. *Chemosphere* 225–236 (2019) doi:10.1016/j.chemosphere.2019.04.047.
52. Hillman, A. L., Yao, A., Abbott, M. B. & Bain, D. J. Two millennia of anthropogenic landscape modification and nutrient loading at Dian Lake, Yunnan Province, China. *Holocene* **29**, 505–517 (2019).
53. Hillman, A. L., Abbott, M. B., Yu, J., Bain, D. J. & Chiou-Peng, T. Environmental legacy of copper metallurgy and mongol silver smelting recorded in yunnan lake sediments. *Environ. Sci. Technol.* **49**, 3349–3357 (2015).
54. Lee, C. S. L. & Qi, S. Seven Thousand Years of Records on the Mining and Utilization of Metals from Lake Sediments in Central China. **42**, 4732–4738 (2008).
55. Hillman, A. L. *et al.* Rapid environmental change during dynastic transitions in

Yunnan Province, China. *Quat. Sci. Rev.* **98**, 24–32 (2014).
